# Balancing Reproduction and Survival: Seasonal Body Mass Dynamics in a High-Altitude Primate (*Rhinopithecus bieti*)

**DOI:** 10.3390/ani16111603

**Published:** 2026-05-25

**Authors:** Yan-Peng Li, Zhi-Pang Huang, Cyril C. Grueter, Xiao-Bin He, Ru-Liang Pan, Xin-Ming He, Gui-Wei Yang, Hua Wu, Liang-Wei Cui, Wen Xiao

**Affiliations:** 1 Institute of Eastern-Himalaya Biodiversity Research, Dali University, Dali 671003, China; liyp@eastern-himalaya.cn (Y.-P.L.); huangzp@eastern-himalaya.cn (Z.-P.H.); 2 School of Life Sciences, Central China Normal University, Wuhan 430079, China; 3 International Center of Biodiversity and Primate Conservation, Dali University, Dali 671003, China; cyril.grueter@anthro.ox.ac.uk (C.C.G.); ruliang.pan@uwa.edu.au (R.-L.P.); 4 Department of Anatomy, Physiology and Human Biology, School of Human Sciences, The University of Western Australia, Perth, WA 6009, Australia; 5 Institute of Human Sciences, School of Anthropology and Museum Ethnography, University of Oxford, Oxford OX1 2JD, UK; 6 Centre of Excellence in Biodiversity and Natural Resource Management, University of Rwanda, Kigali P.O. Box 4285, Rwanda; 7 Centre for Evolutionary Biology, School of Biological Sciences, The University of Western Australia, Perth, WA 6009, Australia; 8 Administration of Baimaxueshan National Nature Reserve, Diqing 674500, China; 9 Administration of Gaoligongshan National Nature Reserve in Nujiang, Nujiang 673200, China; ygw918@foxmail.com; 10 Key Laboratory of Minimal Population Wildlife Conservation in Colleges and Universities of Yunnan Province, Southwest Forestry University, Kunming 650224, China; cuilw@eastern-himalaya.cn

**Keywords:** *Rhinopithecus bieti*, seasonal body mass fluctuation, activity time-budget, reproductive effort, environmental stress, life-history trade-offs

## Abstract

Seasonal body mass fluctuations in mammals reflect fundamental trade-offs between ecological constraints and reproductive effort, yet few studies have simultaneously linked these dynamic changes with activity budgets in high-altitude primates inhabiting extreme-temperature environments. We examined the effects of environmental stress and mating effort on body mass dynamics in black-and-white snub-nosed monkeys (*Rhinopithecus bieti*). Using monthly non-invasive monitoring over a full annual cycle, we obtained 464 body mass records from free-ranging adults (127 male records, 337 female records). Both sexes showed marked seasonal variation: body mass peaked in spring and autumn, declined moderately in winter (males: −7.4%, females: −2.9%), and dropped more sharply during the mating season (males: −11.1%, females: −12.9%). Male mass loss was positively correlated with the number of mates. Seasonal shifts in activity budgets accompanied these patterns: in summer, males increased movement and females increased both movement and social interaction, while both sexes reduced resting; in winter, both sexes increased feeding time, with females additionally reducing social activities. These findings reveal two distinct phases of body mass loss. Body mass decline was more pronounced during the mating season than in winter, a pattern that may be associated with higher energetic costs related to reproductive activity. Our study demonstrates how high-altitude primates balance survival and reproduction through flexible energy allocation, offering key insights into their seasonal energy allocation resilience in provisioned high-altitude habitats.

## Simple Summary

Many wild animals living in high-altitude areas face harsh seasonal changes, including cold winters and limited food. How they balance survival and reproduction is key to their adaptation. We studied seasonal body mass changes in black-and-white snub-nosed monkeys, a rare high-altitude primate species, over one full year. We recorded body mass and daily behaviors to understand energy use. Our results show that both male and female monkeys lost body mass in winter and during the mating season, with greater loss during mating. Males lost more weight when they had more female mates. In the mating season, monkeys moved more and rested less; in winter, they spent more time feeding. These patterns suggest mating activity uses more energy than winter environmental stress. This study helps explain how high-altitude primates cope with seasonal challenges. The findings improve public and scientific understanding of rare primate adaptation and support better conservation management for this endangered species.

## 1. Introduction

Seasonal variation in body mass is a central feature of mammalian ecology, reflecting fundamental trade-offs between energy acquisition and expenditure [1,2]. Fluctuations in body mass often track seasonal dynamics in energy and nutrient intake, which are shaped by shifts in the abundance and quality of food resources [3,4,5,6]. In highly seasonal montane-temperate environments, predictable changes in food availability and extreme temperature conditions impose recurrent nutritional bottlenecks [7,8]. During periods of cold stress, basal metabolic rate increases, thermoregulatory costs rise, and foraging efficiency declines, collectively imposing substantial energetic demands on resident animals [9,10]. For instance, Tibetan macaques (*Macaca thibetana*) at Mount Emei reach their annual peak body mass in autumn before entering the energetically demanding winter, during which females lose up to 32% of body mass and males more than 13% [11]. Similarly, Sichuan snub-nosed monkeys (*Rhinopithecus roxellana*) in north-temperate forests exhibit marked winter mass loss in response to cold stress and food scarcity [12]. Many species attempt to mitigate these challenges by adjusting their activity budgets, often increasing feeding time to compensate for the decline in food quality during the lean season [12,13,14,15].

Beyond ecological constraints, reproduction imposes an additional layer of energetic costs. Life-history theory predicts that allocation of energy toward reproduction inevitably comes at the expense of survival, producing measurable trade-offs in body condition and reproductive behavior [16,17]. In polygynous primates, these trade-offs are especially pronounced. Males invest heavily in mate competition, including guarding, territorial defense, and courtship, often resulting in significant body mass loss during breeding periods [18,19,20,21,22,23,24]. Females likewise face energetic demands from mating competition, pregnancy, and lactation, leading to marked seasonal fluctuations in body condition [24,25,26,27,28]. Empirical studies illustrate these principles across diverse taxa. In Hanuman langurs (*Semnopithecus entellus*), conceptions were concentrated during periods of best female physical condition, suggesting that nutrition influences reproductive timing and conception probability [29]. Male chimpanzees (*Pan troglodytes*) also incur energetic costs, sacrificing foraging time for coalitionary aggression and mate guarding [30]. Female Japanese macaques (*Macaca fuscata*) undergo body mass decline at the end of the mating season, reflecting the costs of sexual solicitation and reproductive activity [27]. Together, these findings highlight that seasonal body mass fluctuations are not solely responses to ecological pressures, but also reflect reproductive investment.

The black-and-white snub-nosed monkey (*R. bieti*, Colobinae) offers an ideal system to study the intersection of these two pressures. Endemic to the montane forests of the Yunnan-Guizhou Plateau in southwest China, *R. bieti* inhabits elevations of 2600–4600 m, where snow persists for much of the year and annual mean temperatures remain below 10 °C [31,32]. Seasonal dietary shifts are pronounced: lichens dominate in winter, young leaves in spring, bamboo shoots in summer, and fruits in autumn [7,33,34,35]. The scarcity of high-quality food during winter presents a major ecological challenge, requiring adaptive strategies for energy storage and allocation. Compounding these ecological stresses, *R. bieti* exhibits a complex multilevel social system characterized by one-male multi-female units (OMUs) embedded in larger bands, alongside peripheral all-male units (AMUs) [32,36]. Its polygynous mating system, with high female-to-male ratios (typically 3:1, up to 5:1), generates intense intra- and inter-sexual competition [37]. Notably, this species exhibits marked sexual size dimorphism, with adult males weighing approximately twice as much as adult females, a trait linked to intense male–male competition [38]. Resident males must not only guard mates within their OMU but also repel challenges from extra-unit males attempting takeovers or copulations [39]. Females, in turn, actively engage in sexual solicitation, intra-sexual competition, and grooming to maximize reproductive success [40]. Thus, *R. bieti* simultaneously experiences strong ecological stressors and high reproductive competition, making it a compelling model for investigating how primates balance survival and reproduction in high-altitude and strongly seasonal environments.

In this study, we investigate seasonal body mass fluctuations in free-ranging *R. bieti* by integrating monthly non-invasive body mass monitoring with detailed activity budget data across an entire annual cycle. This approach allows us to directly link body mass variation with ecological stress and reproductive effort. Based on the dual pressures of environmental constraints and mating competition, we propose two sets of hypotheses:

**Hypothesis** **1 (Winter energetic stress).**
*Cold temperatures and food scarcity in winter cause significant body mass loss.*

**Hypothesis** **H1a.**
*Individuals exhibit measurable body mass loss in winter.*

**Hypothesis** **H1b.**
*Activity budgets are adjusted in winter to mitigate the extent of body mass loss.*

**Hypothesis** **2 (Reproductive energetic costs).**
*Mating competition imposes sex-specific energy expenditure.*

**Hypothesis** **H2a.**
*Adult males lose body mass after the mating season.*

**Hypothesis** **H2b.**
*Male body mass loss is positively correlated with the number of females in their OMU.*

**Hypothesis** **H2c.**
*Adult females also experience significant body mass loss after the mating season.*

By linking body mass fluctuations to seasonal variation in activity allocation and reproductive dynamics, this study sheds light on the fundamental trade-offs between survival and reproduction. These findings also advance our understanding of energy allocation strategies in seasonal environments.

## 2. Materials and Methods

### 2.1. Study Site and Subjects

This study is part of a long-term research project on the behavior and ecology of black-and-white snub-nosed monkeys living in the Shangri-la Yunnan Golden Monkey National Park (27°30′ N, 99°20′ E) in the Baimaxueshan National Nature Reserve. The vegetation at the study site is composed predominantly of mixed conifer and broad-leaf forest.

All adult individuals in the study group were fully habituated and individually identifiable. On 15 April 2012, the study group comprised 87 individuals which were associated with a total of eight OMUs (one-male multi-female unit) and one AMU (all-male unit). Units ranged in size from 3 to 13 individuals. The adults sex ratio of the study group (excluding the AMU) was approximately 1 male: 2.5 females (Table 1). We focused on adult individuals in this study because the body mass of immature individuals changes during ontogenetic development [38]. Males were considered adult and sexually mature when they were more than eight years old and females when they were more than five years old [38].

To attract monkey individuals to the study site, the park rangers provisioned them with a small number of supplementary foods (30 kg of *Usnea longissima* and 10 kg of Sumac seeds) every single day of the whole year. Lichens were evenly scattered across the provisioning site by staff in the morning. Sumac seeds were distributed by age/sex: approximately 200 g for young individuals, 300 g for adult females, and 400 g for adult males. Each unit is managed by a dedicated staff to avoid food being completely occupied by one high ranking individual. The monkeys’ overall dietary intake consisted of 28% supplementary foods and 72% natural foods. During behavioral data collection, we quantified dietary composition by recording the proportion of natural versus artificial food sources consumed. We quantified dietary intake by calculating the proportion of total feeding time allocated to each food category during focal observations. We also provided some attractive foods such as a few slices of apple or a carrot to lure the monkeys onto the scale for body mass measuring; the weight of these foods did not exceed 50 g per individual, the bait food (apple/carrot) was excluded from body mass measurements by resetting the scale after placing the bait on it. The weight of these foods was subtracted from the body mass measurements by resetting the scale after placing the food on it.

### 2.2. Season Division

**Figure 1:**
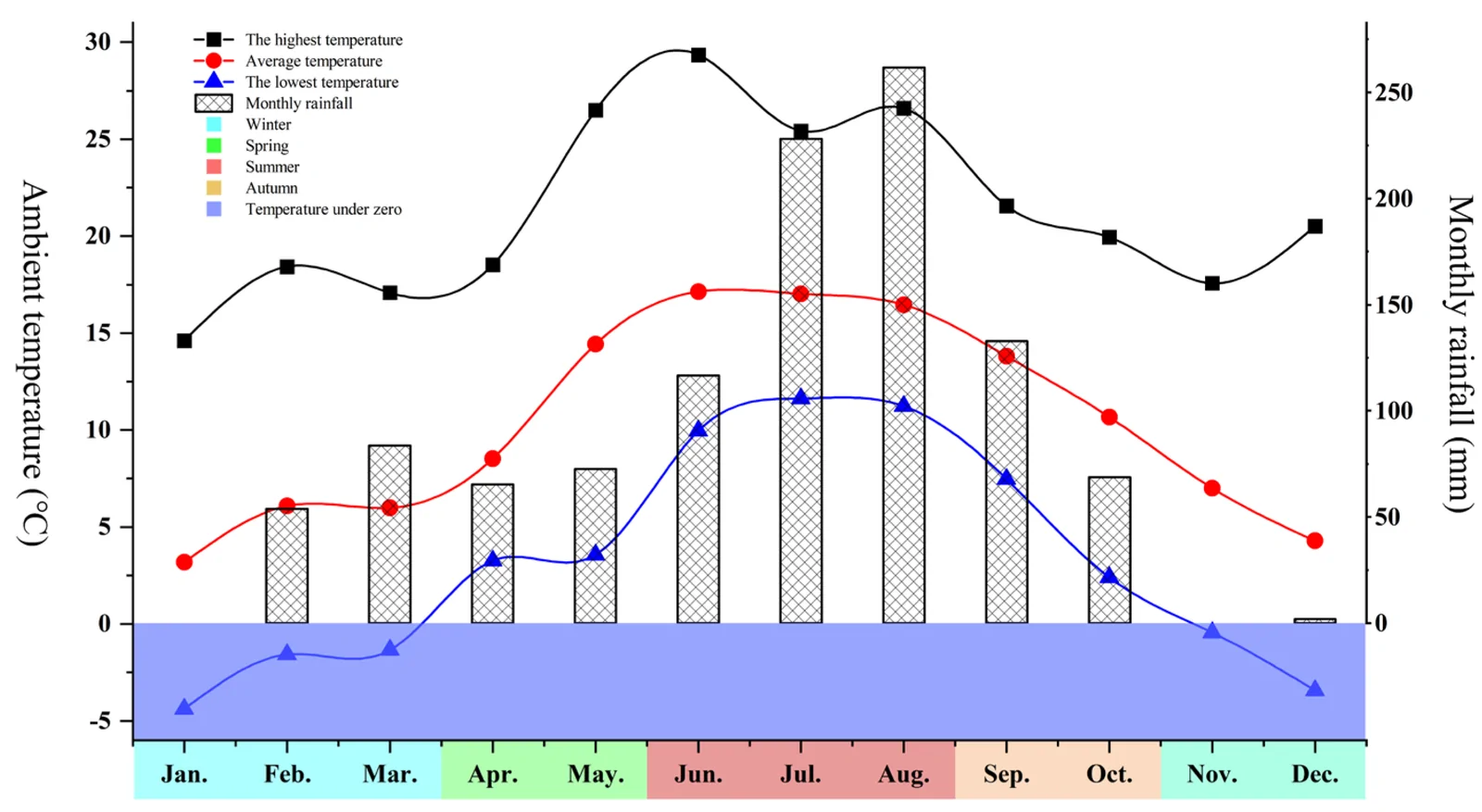
Ambient temperature (in degrees Celsius) and rainfall across seasons. The colored lines indicate maximum, minimum, and average temperatures in the Shangri-la Yunnan Golden Monkey National Park, China. The bars show the monthly rainfall volume in mm.

We used a meteorological monitoring system (HOBO RG-3M, Onset Computer Corporation, Bourne, MA, USA) located at 2800 m to record the air temperature (with an accuracy of 0.1 °C, recorded once per 30 min) and precipitation (with an accuracy of 0.2 mm). The average annual temperature was 10.38 °C and total rainfall over a one-year period was 1085.40 mm. We divided the year into four seasons (Figure 1) according to China’s Meteorological Administration rule [41]. We chose meteorological season division rather than biological markers (e.g., reproductive state) because seasonal changes in the study site are highly synchronized with temperature fluctuations, and this method allows for direct comparison with other temperate primate studies.

Winter: Starts when the daily average temperature is below 10 °C for 5 days and ends when the daily average temperature exceeds or equals 10 °C for 5 days. According to these criteria, winter at the site lasts for more than five months (from November to March of the following year).

Summer: Starts when the daily temperature exceeds 22 °C for 5 days and ends when the daily temperature is equal to or less than 22 °C for 5 days. Summer at the study site lasts approximately two months (June to August).

Spring: The period between winter and summer (April and May).

Autumn: The period between summer and winter (September and October).

Mating period: Black-and-white snub-nosed monkeys show a seasonal peak in mating activity which corresponds to the mating season. The exact timing of the mating season varies across locales: June to August in a northern population in Mangkang [42], July to September in a southern population in Lanping [43] and August to October in a captive population in Kunming [44]. Our study population is geographically close to the northern part of the species’ distribution range, with a mating season lasting from June to August. Another way to determine the extent of the mating season is to use data on gestation length and timing of births. With a gestation length of 195–200 days [45] and 83.1% of newborn infants (recorded in the months of February, March and April, the corresponding mating season would fall into the period from June to August.

### 2.3. Body Mass Measurements

**Figure 2:**
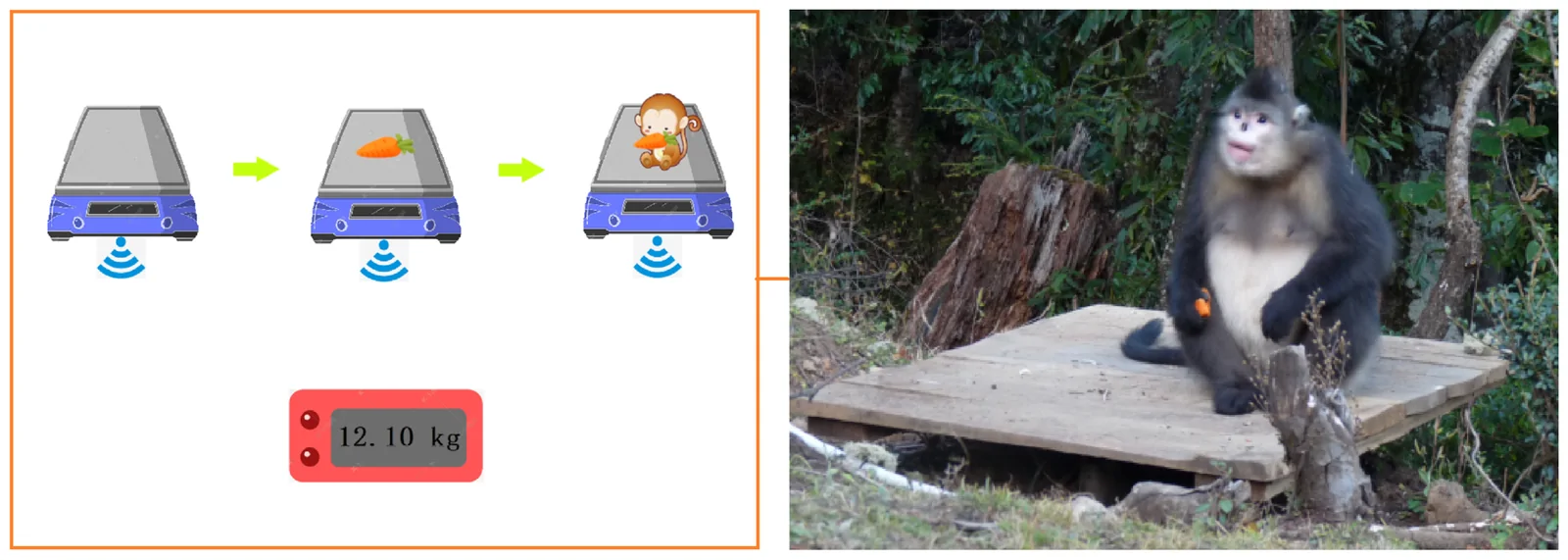
Monkey weighing process at the study site. Before the monkeys traveled to the feeding site in the afternoon, a digital scale was placed on a relatively flat place and their favorite food was put on the scale. When a single individual stepped onto the scale, we recorded its body mass together with information about its identity (age, sex, OMU, etc.).

Over a period of 12 consecutive months from March 2012 to February 2013, we collected 464 body mass records, 127 from adult males and 337 from adult females. Body mass data were obtained with a wireless electronic scale (XK3190-A12-E, max value of 100.00 kg, min value of 0.02 kg, ShangHai YaoHua, Shanghai, China), which was explicitly designed for an environment with a relative humidity of 10–85% and a temperature range between −10 °C and 40 °C. Before the monkeys arrived at the feeding site in the afternoon, we placed a wireless electronic scale on a relatively flat place at the feeding site. We obtained body mass data when a single monkey went to eat the foods that had been placed on the scale. We also recorded the identity, age, and sex of the individual and the social unit (Figure 2). Some individuals could be weighed more than once in the same month; in these cases, we took the arithmetic mean value, with an average of 3.2 ± 1.1 repeat measurements per individual per month (range: 1–6).

### 2.4. Behavioral Data Collection

From March 2012 to February 2013, we observed the study subjects’ behavior using instantaneous scan sampling with a 5 min interval between scans; at each predetermined time point, we conducted instantaneous scan sampling to record the presence (1) or absence (0) of each behavioral state for all visible individuals. The time allocated to each behavior was calculated as the proportion of scans in which that behavior was observed, relative to the total number of valid scans. Each behavior was coded (F, R, M, G) and counted to derive activity budgets [46,47]. Behavioral data were collected from 8:00 to 18:30 on an average of ten days each month (range: 5–24 days). The total number of observation days was 124 (16,005 sample records). Behavioral data were collected for the purpose of calculating time budgets. Behaviors were classified into four categories: feeding, resting, moving, and social behavior. Feeding included all behaviors related to food acquisition, selection, handling (peeling, sorting, chewing), and ingestion. Resting was defined as a state of complete inactivity, during which individuals remained stationary, often with a slightly lowered head, downward-facing face, or closed eyes. Moving referred to the displacement of an individual from one location to another, including climbing, leaping, and swinging. Social behavior included all inter-individual interactions such as grooming, fighting, playing, threatening, sexual solicitation, and copulation [48]. Notably, activity type is used as a proxy for energy expenditure in this study, consistent with standard primate energetics research [30].

### 2.5. Statistical Analyses

We did not run any statistics on the body mass of females who became pregnant (3 individuals) during the mating season (i.e., those who were found to give birth in the subsequent birth season), because body mass changes in pregnant females during winter would change our understanding of the natural fluctuations under cold stress.

To analyze the effect of season on body mass, we fitted two Generalized Linear Mixed Models (GLMMs) with Gaussian error structure and identity link function, one for adult males and one for adult females. Body mass was modeled as the dependent variable. The fixed effect was season (factor with four levels). To factor in the potentially disproportionate effect of certain study subjects on the dependent variable, we included the identity of the weighed animal as a random effect. We checked for the assumptions of normally distributed and homogeneous residuals by visually inspecting the distribution of the residuals and plotting the residuals against fitted values. Models were fitted with Restricted Maximum Likelihood [49]. We used an unadjusted Tukey test to derive *p*-values for the pairwise comparisons of levels of the categorical predictor. We calculated marginal (variance explained by the fixed effects) and conditional (variance explained by the entire model, including both fixed and random effects) coefficients of determination following Nakagawa and Schielzeth [50] and Johnson [51].

GLMMs was fitted in R version 4.3.3 [52] using the function ‘lmer’ of the package lme4 (version 1.1–35.3) [53]. Confidence intervals were computed using the function ‘confint’. R^2^ was calculated using the function GLMM’ from the R package MuMIn (version 1.19.13) [54]. We used the lmerTest package (version 2.0-36) [55] to calculate *p*-values for fixed effects using Satterthwaite’s approximation for degrees of freedom. We performed pairwise multiple comparisons among season levels using the glht function in the multcomp package (version 1.4-30).

The body mass loss rate was calculated using the following formula:

R = L/W × 100

where R is the body mass loss rate; L is the lost body mass; and W is the body mass recorded in November (the first month of winter) or June (the first month of the mating season).

We further used GLMMs to test body mass shifts associated with two key biological periods: (1) before versus after the mating season, and (2) before versus after winter. These models included sex and period as fixed factors, with individual identity and OMU identity as random effects to account for repeated measures and potential among-group variation. These models were designed to directly evaluate our core hypotheses regarding mating effort and winter energetic stress, and no seasons were pooled into a combined “other seasons” category. To examine whether reproductive competition affected male condition, we used linear regression to test the relationship between body mass loss of unit leader males (continuous variable) and the number of females in their breeding units (continuous variable). Non-parametric alternatives (Mann–Whitney U test) were additionally checked to ensure robustness of results given the categorical predictors.

We calculated daily time budgets for different activities for males and females separately. Our behavioral sample included 123 days’ worth of data for adult males (52 days from the mating season and 47 days from winter) and 124 days for adult females (52 days from mating season and 47 days from winter). We divided the number of records of each behavior by the total number of records of all behaviors in each observation period (one day), as replicates. Due to the non-normal distribution of data, we compared behaviors performed in different seasons using the Two-Independent-Samples Kolmogorov–Smirnov test, between the mating season and other seasons and between winter and other seasons, with significant levels (2-tailed) set at *p* < 0.05 (significant at the 0.05 level), *p* < 0.01 (highly significant at the 0.01 level), and *p* < 0.001 (extremely significant at the 0.001 level).

## 3. Results

### 3.1. Effect of Season on Body Mass

**Figure 3:**
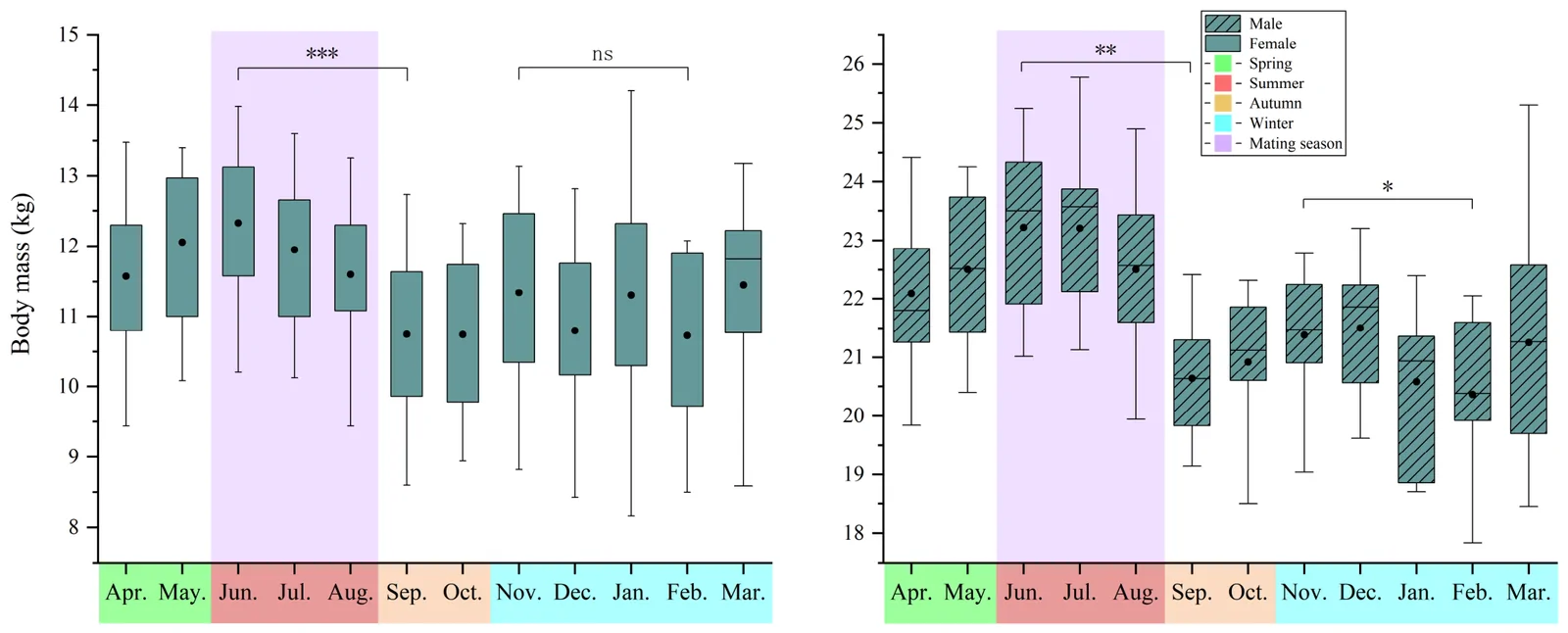
Monthly body mass changes of adult black-and-white snub-nosed monkeys over a full annual cycle. Boxplots show mean (●), inter-quartile range (box) and maximum and minimum values (whiskers). Purple background = mating season. The number of male individuals sampled was: 9 in January, 8 in February, 15 in March, 10 in April, 9 in May, 8 in June, 9 in July, 8 in August, 8 in September, 9 in October, 8 in November, 11 in December. The number of female individuals sampled was 17 in January, 15 in February, 20 in March, 24 in April, 24 in May, 21 in June, 21 in July, 22 in August, 19 in September, 16 in October, 16 in November, 15 in December. n.s. indicates there was no significant seasonal difference fora particular activity (*p* > 0.05), * indicates significant seasonal differences at *p* < 0.05, ** indicates significant seasonal differences at *p* < 0.01, *** indicates significant seasonal differences at *p* < 0.001.

Both males and females exhibited clear seasonal fluctuations in body mass. In the male model, the marginal variance was 0.18 and the conditional variance was 0.79, while in the female model, the marginal variance was 0.09 and the conditional variance was 0.68. The random effect (individual variation) contributed variances of 2.24 (SD: 1.46) for males and 1.20 (SD: 1.10) for females (Table 2). Individual identity explained a substantial proportion of variance in both sexes. Males reached their maximum average body mass in June (23.22 ± 1.52 kg) and the minimum in February (19.80 ± 1.49 kg), while females peaked in June (12.33 ± 1.38 kg) and reached their lowest body mass in February (10.73 ± 1.17 kg; Figure 3).

### 3.2. Body Mass Loss

During the long cold winter (November to February), males experienced an average body mass loss of 7.4% (1.58 kg; mean: 21.39 ± 0.42 kg in November vs. 19.80 ± 0.58 kg in February, N_Nov_ = 8, N_Feb_ = 8; GLMM: estimate = 1.45, SE = 0.60, t = 2.40, *p* = 0.046). In contrast, non-pregnant females showed a smaller average loss of 2.9% (0.32 kg; mean: 11.05 ± 0.34 kg in November vs. 10.73 ± 0.30 kg in February, N_Nov_ = 18, N_Feb_ = 15; GLMM: estimate = 0.47, SE = 0.34, t = 1.38, *p* = 0.189), which was not statistically significant (Figure 3).

Significant body mass loss was observed in both sexes following the mating season (summer, June to September). Females lost an average of 1.59 kg, corresponding to 12.9% of their body mass (mean: 12.33 ± 0.28 kg in June vs. 10.75 ± 0.26 kg in September, N_Jun_ = 21, N_Sep_ = 19; GLMM: estimate = −1.55, SE = 0.27, t = −5.16, *p* < 0.0001). Males exhibited an average loss of 2.58 kg, corresponding to 11.1% of their body mass (mean: 23.22 ± 0.54 kg in June vs. 20.64 ± 0.39 kg in September, N_Jun_ = 8, N_Sep_ = 8; GLMM: estimate = −2.52, SE = 0.49, t = −5.16, *p* = 0.001) (Figure 3).

**Figure 4:**
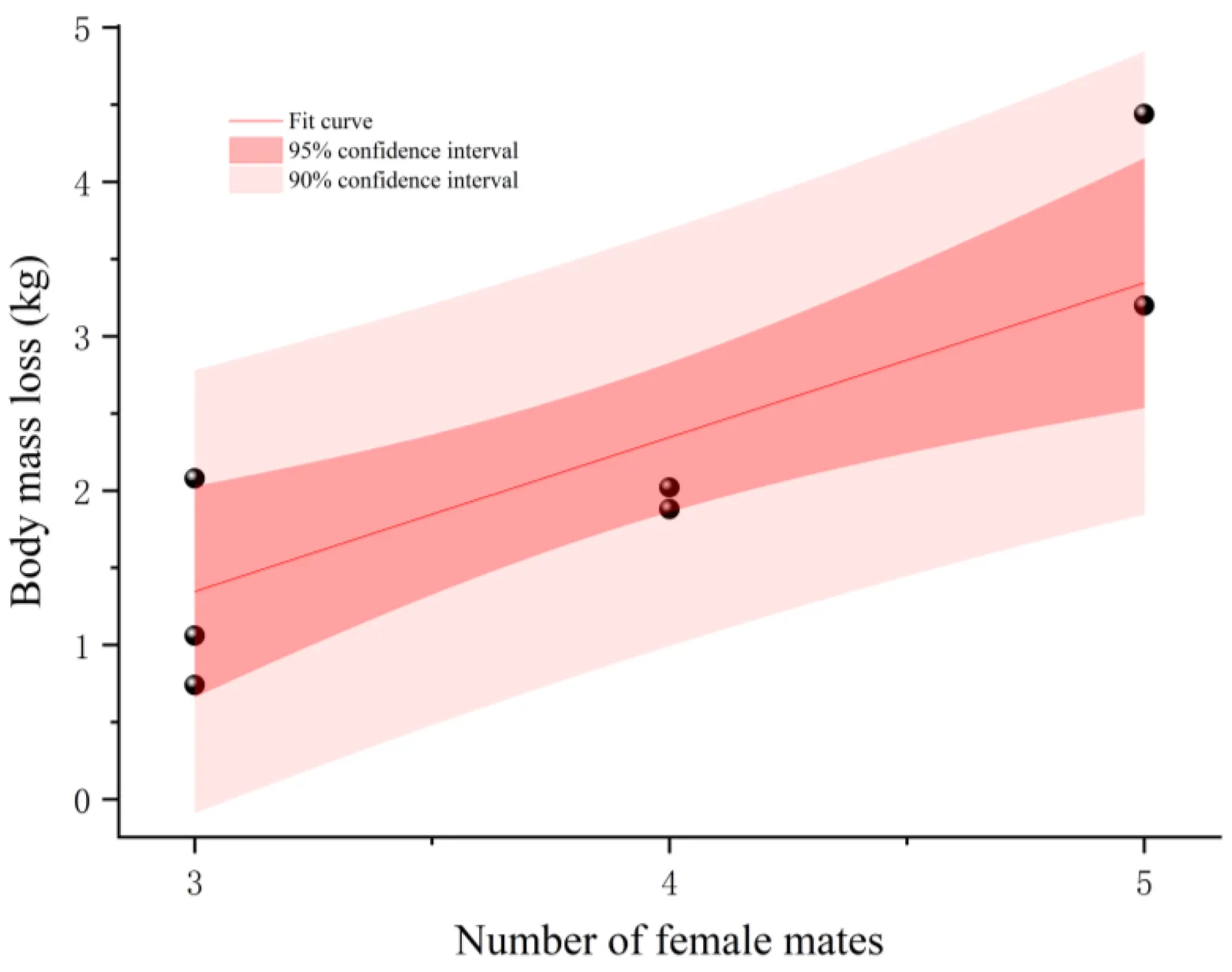
Linear relationship between male body mass loss during the mating season and number of female mates in their breeding units.

There was a significant linear relationship between male body mass loss and the number of its female mates in their breeding units y = 1.228x − 2.532 (y = body mass loss, x = the number of its female mates, r^2^_adj_ = 0.715, F = 16.070, *p* < 0.01; Figure 4).

### 3.3. Body Mass Recovery

During the spring (March to June), adult males experienced a significant average body mass gain of 11.26% (2.35 kg), increasing from 20.87 ± 0.057 kg in March to 23.22 ± 0.71 kg in June (N_Mar_ = 16, N_Jun_ = 8; GLMM: estimate = 1.753, SE = 0.714, t = 3.286, *p* < 0.001). In comparison, non-pregnant females exhibited a significant gain of 7.65% (0.876 kg), with their body mass increasing from 11.45 ± 0.287 kg in March to 12.33 ± 0.33 kg in June (N_Mar_ = 20, N_Jun_ = 21; GLMM: estimate = 0.876, SE = 0.33, t = 2.654, *p* = 0.008).

Body mass gain was observed in both sexes following autumn (September to November). Females exhibited an average gain of 5.50% (0.591 kg), increasing from 10.75 ± 0.26 kg in September to 11.34 ± 0.31 kg in November (N_Sep_ = 19, N_Nov_ = 16; GLMM: estimate = 0.591, SE = 0.353, t = 1.677, *p* = 0.094). Males exhibited an average gain of 3.63% (0.75 kg), increasing from 20.64 ± 0.39 kg in September to 21.39 ± 0.42 kg in November (N_Sep_ = 8, N_Nov_ = 8; GLMM: estimate = 0.75, SE = 0.082, t = 9.165, *p* < 0.001).

### 3.4. Activity Time Budget During the Mating Season

**Figure 5:**
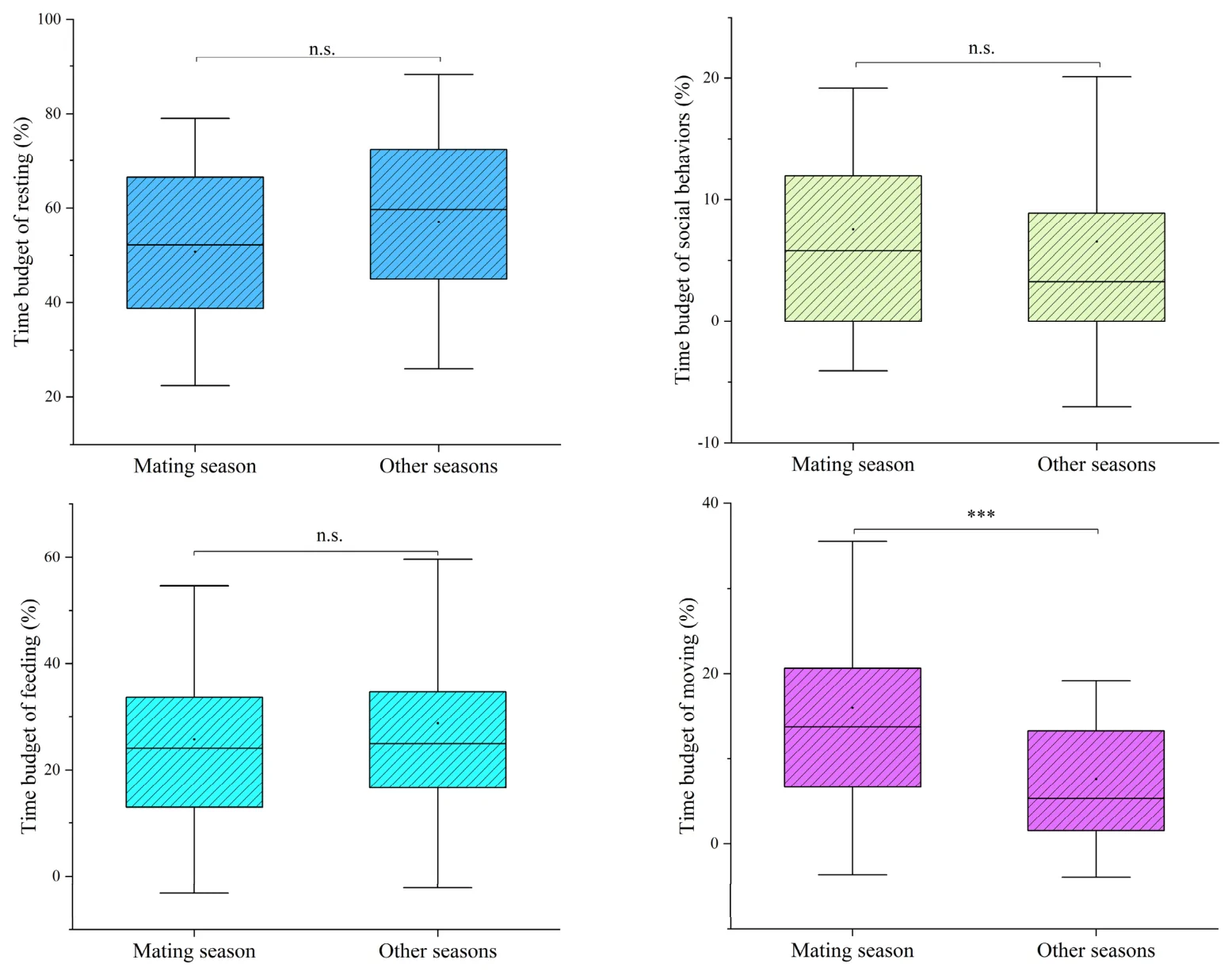
Time budget of male snub-nosed monkeys during the mating season and other seasons. n.s. indicates there was no significant seasonal difference fora particular activity (*p* > 0.05), *** indicates significant seasonal differences at *p* < 0.001.

Adult males significantly increased their time spent moving in the mating season compared to other seasons (mean value: 15.97%_mating_ ± SE 1.81% vs. 8.10%_other_ ± SE 0.92%, N_mating_ = 52, N_other_ = 71, t = 4.172, df = 121, *p* < 0.001). They also increased their time investment in social behaviors (mean value: 7.56%_mating_ ± SE 1.08% vs. 6.69%_other_ ± SE 1.08%, N_mating_ = 52, N_other_ = 71), but this was not statistically significant (t = 0.558, df = 121, *p* = 0.578). Males reduced their time budget for feeding and resting in the mating season (feeding mean value: 25.73%_mating_ ± SE 2.67% vs. 28.32%_other_ ± SE 2.51%, N_mating_ = 52, N_other_ = 71, t = 0.695, df = 121, *p* = 0.488; resting mean value: 50.74%_mating_ ± SE 2.62% vs. 56.90%_other_ ± SE 2.52%, N_mating_ = 52, N_other_ = 71, t = 1.665, df = 121, *p* = 0.099), but this was not statistically significant (Figure 5).

**Figure 6:**
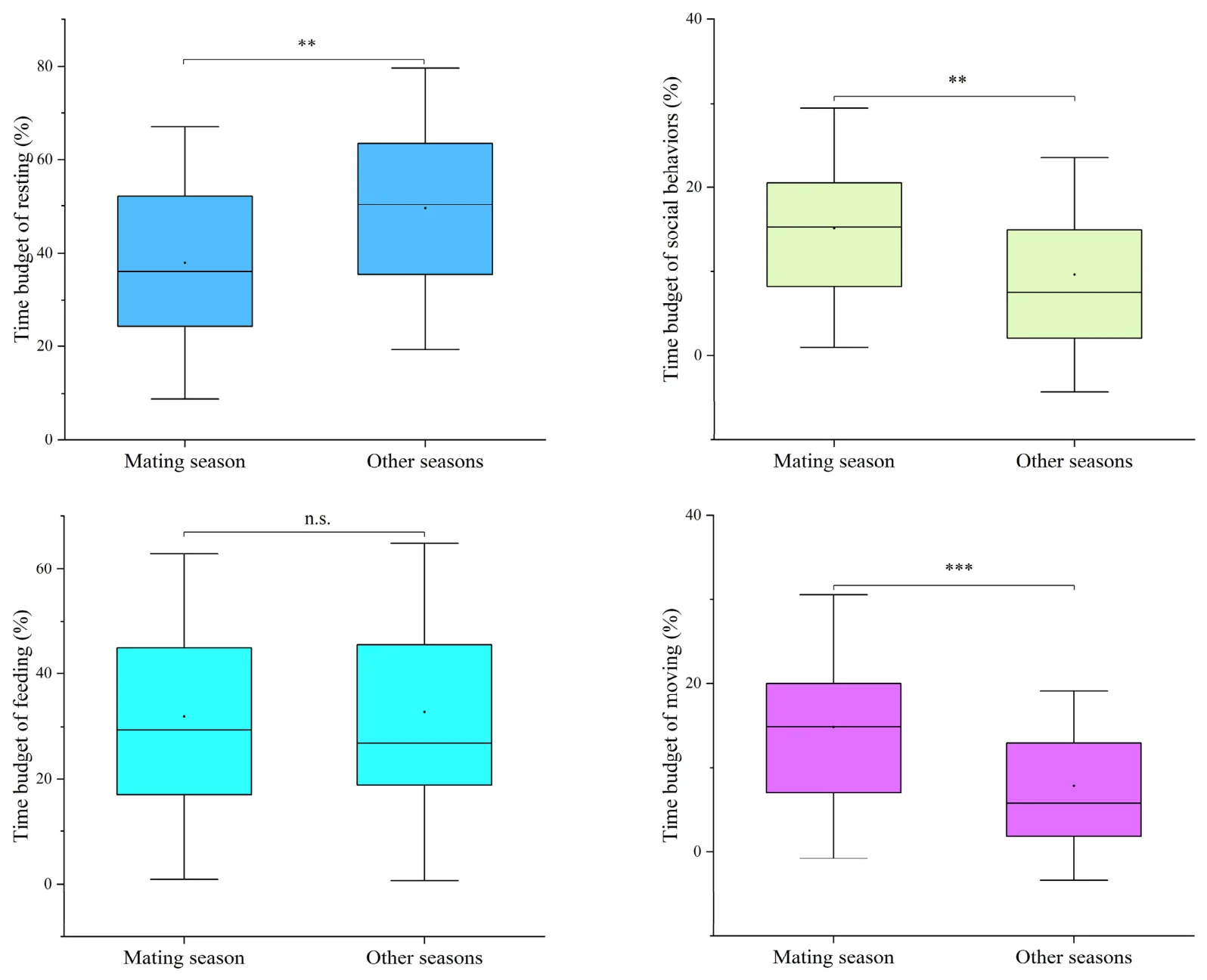
Time budget of female snub-nosed monkeys during the mating season and other seasons. n.s. indicates there was no significant seasonal difference fora particular activity (*p* > 0.05), ** indicates significant seasonal differences at *p* < 0.01, *** indicates significant seasonal differences at *p* < 0.001.

Adult females significantly reduced their time spent resting in the mating season (mean value: 37.99%_maring_ ± SE 2.69% vs. 48.96%_other_ ± SE 2.40%, N_mating_ = 52, N_other_ = 72, t = 3.02, df = 122, *p* = 0.003). They also showed a significant increase in time allocated to moving and social behaviors (moving mean value: 14.92%_winter_ ± SE 1.45% vs. 8.24%_other_ ± SE 0.89%, N_mating_ = 52, N_other_ = 72, t = 4.126, df = 122, *p* < 0.001; social behavior mean value: 15.19%_mating_ ± SE 1.32% vs. 10.14%_other_ ± SE 1.17%, N_mating_ = 52, N_other_ = 72, t = 2.845, df = 122, *p* = 0.005). Females devoted less time to feeding in the mating season (mean value: 31.90%_mating_ ± SE 2.86% vs. 32.66%_other_ ± SE 2.57%, N_mating_ = 52, N_other_ = 72), but this was not statistically significant (t = 0.194, df = 122, *p* = 0.847) (Figure 6).

### 3.5. Activity Time Budget in Winter

**Figure 7:**
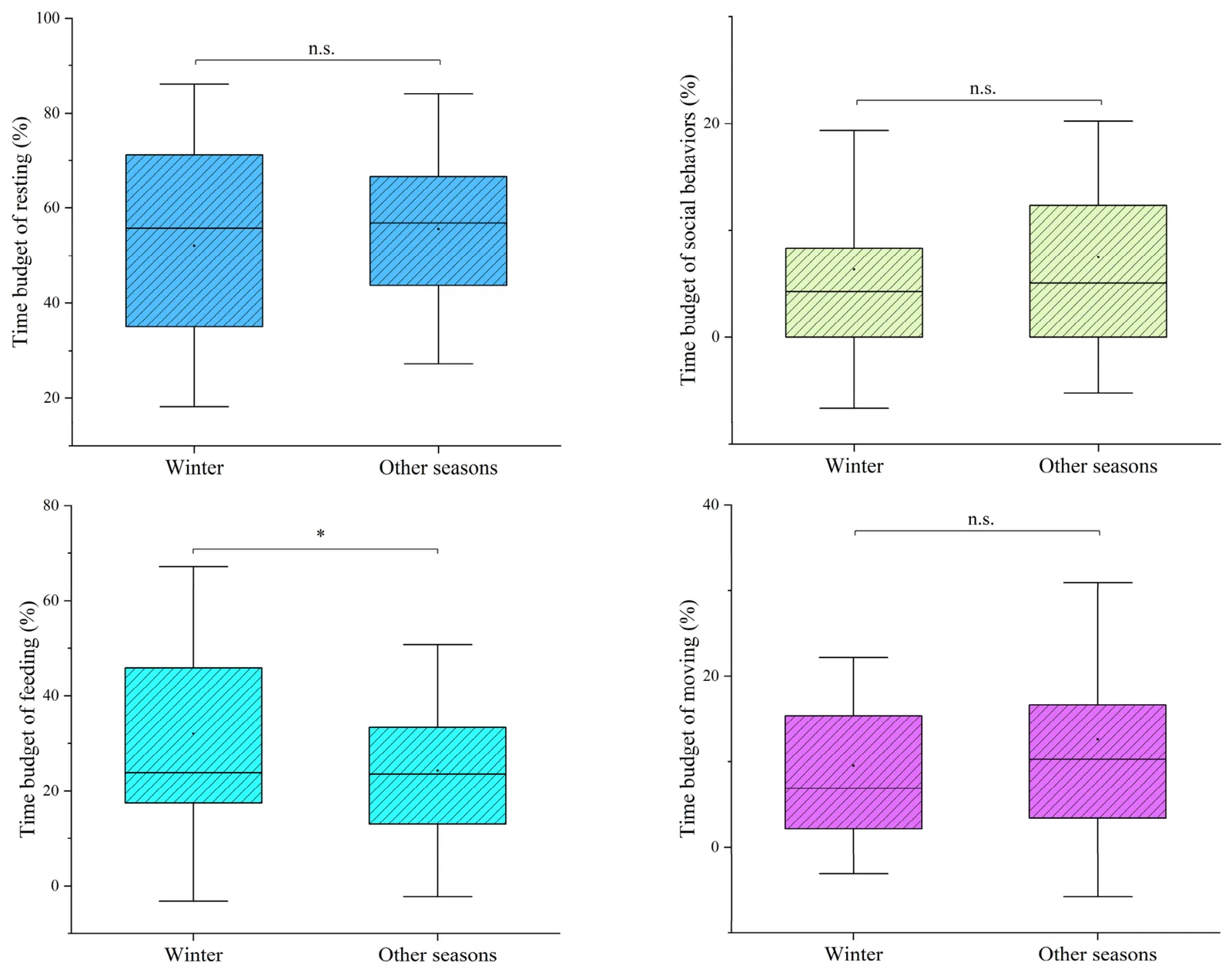
Time budget of male snub-nosed monkeys during winter and other seasons. n.s. indicates there was no significant seasonal difference fora particular activity (*p* > 0.05), * indicates significant seasonal differences at *p* < 0.05.

Adult males allocated significantly more of their time to feeding (mean value: 31.99%_winter_ ± SE 3.42% vs. 24.27%_other_ ± SE 2.02%, N_winter_ = 47, N_other_ = 76, t = 2.074, df = 121, *p* = 0.040) in winter compared to other seasons. They reduced their time engaged in resting, moving and social behaviors in winter compared to other seasons (resting mean value: 52.13%_winter_ ± SE 3.30% vs. 55.63%_other_ ± SE 2.17%, N_winter_ = 47, N_other_ = 76, t = 0.924, df = 121, *p* = 0.357; moving mean value: 9.53%_winter_ ± SE 1.23% vs. 12.60%_other_ ± SE 1.40%, N_winter_ = 47, N_other_ = 76, t = 1.514, df = 121, *p* = 0.133; social behavior mean value: 6.35%_winter_ ± SE 1.26% vs. 7.49%_other_ ± SE 0.098%, N_winter_ = 47, N_other_ = 76, t = 0.720, df = 121, *p* = 0.473), but these changes were not statistically significant (Figure 7).

**Figure 8:**
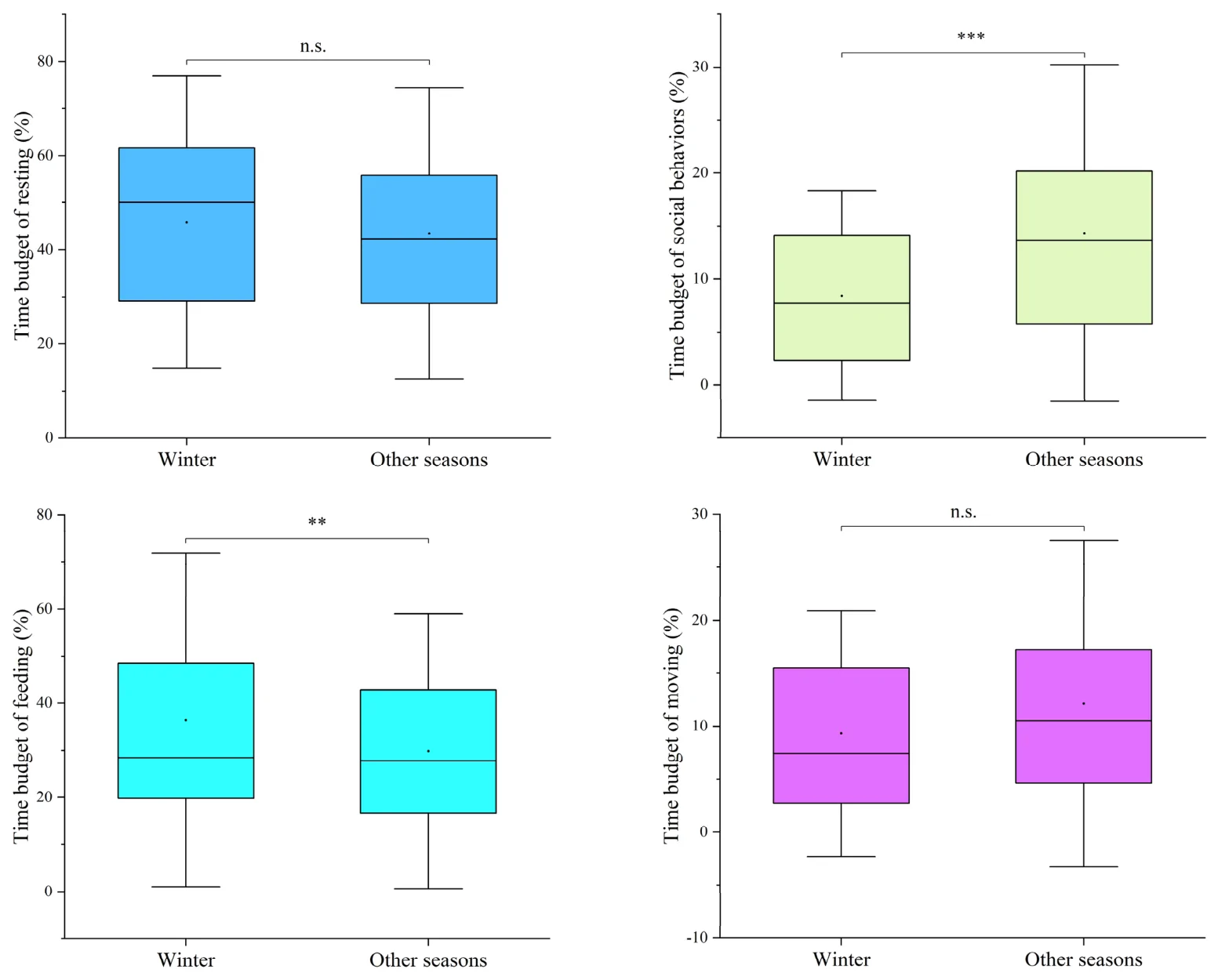
Time budget of female snub-nosed monkeys during winter and other seasons. n.s. indicates there was no significant seasonal difference fora particular activity (*p* > 0.05), ** indicates significant seasonal differences at *p* < 0.01, *** indicates significant seasonal differences at *p* < 0.001.

Adult females significantly increased their feeding in winter (mean value: 36.44%_winter_ ± SE 3.44% vs. 29.84%_other_ ± SE 2.22%, N_winter_ = 47, N_other_ = 77, t = 1.960, df = 122, *p* = 0.0094). They also increased their time budget for resting (mean value: 45.83%_winter_ ± SE 3.02% vs. 43.47%_other_ ± SE 2.35%, N_winter_ = 47, N_other_ = 77, t = 618, df = 122, *p* = 0.538), but this was not statistically significant. Females significantly reduced their time spent socializing in winter (mean value: 8.43%_winter_ ± SE 0.96% vs. 14.59%_other_ ± SE 1.26%, N_winter_ = 47, N_other_ = 77, t = 3.463, df = 122, *p* = 0.0028). They also reduced their time spent moving (mean value: 12.11%_winter_ ± SE 1.17% vs. 9.30%_other_ ± SE 1.13%, N_winter_ = 47, N_other_ = 77, t = 1.618, df = 122, *p* = 0.108), but this change was not statistically significant (Figure 8).

## 4. Discussion

Our findings demonstrate clear seasonal fluctuations in body mass and activity budgets in *R. bieti*, which may be associated with adaptive strategies to balance energetic challenges related to reproduction and survival.

### 4.1. Body Mass Variation and Loss Across the Year

Our results demonstrate marked seasonal fluctuations in the body mass of black-and-white snub-nosed monkeys. Body mass peaked in June, coinciding with the availability of high-quality resources such as young leaves and bamboo shoots in spring [37]. Similarly to rhesus macaques (*Macaca mulatta*) and golden lion tamarins (*Leontopithecus rosalia*), which increase food intake prior to reproduction to build energy reserves [22,56], *R. bieti* likely accumulate surplus energy to cope with the upcoming demands of the mating season.

Following the summer mating season, body mass partially recovered in autumn, but reached the annual minimum in late winter, when monkeys experienced prolonged cold exposure and a severe shortage of preferred foods (Figure 3). Overall, these patterns are consistent with our prediction (H1a, H2a, and H2c) that *R. bieti* undergo significant body mass loss both during winter and the mating season, patterns that may be associated with the dual energetic challenges of survival and reproduction.

### 4.2. Body Mass and Activity Changes During the Cold, Resource-Scarce Season

Both sexes exhibited body mass decline in winter (November–March), although the magnitude was modest compared to other temperate primates in China, such as Tibetan macaques (32% for males, 13% for females) and golden snub-nosed monkeys (14%) [11,12]. The relatively minor loss in *R. bieti* may be attributed to two factors: (1) excess energy intake during autumn through fruits and other calorie-rich foods [12,27,57] and (2) seasonal adjustments in activity budgets [13,58,59], and also group hugging, which can eliminate energy loss [59].

In winter, both males and females increased their feeding time while reducing social behaviors, thereby enhancing energy acquisition and reducing expenditure. Such behavioral plasticity, also reported in other temperate-living species [12,14,60,61,62,63,64], helps mitigate the dual stresses of low temperatures and poor food quality. The ability to digest fibrous lichens-a major winter fallback food-is further supported by a specialized four-chambered stomach that facilitates microbial fermentation and nutrient extraction [65]. These adaptations collectively buffer energy deficits, though they cannot entirely prevent body mass loss.

Anthropogenic factors may also contribute. Provisioning by park staff, although consistent year-round, likely supplements dietary shortages in winter, further dampening seasonal weight loss.

Finally, sex differences in winter body mass loss suggest an additional social dimension. Males lost more weight than females, possibly due to continuous pressure to defend mating rights against rival males, including frequent takeover attempts by all-male units [39]. Such vigilance and competitive investment reduce resting opportunities [66], keeping males energetically taxed even outside the mating season.

### 4.3. Body Mass Changes During the Mating Season

We also predicted that, due to the energetic demands of mating competition, adult males and females in the breeding units would experience body mass loss after the mating season (June to August). This prediction was confirmed by the results. An energy surplus acquired during spring/early summer (see above) can prepare individuals for sustained ‘endurance rivalry’ [67] during the mating season when energy expenditure is significant [22,56]. After the three-month mating season, adult snub-nosed monkey males exhibited a body mass loss of 11.1% (2.58 kg), while females showed an even greater body mass reduction of 12.9% (1.58 kg). Energetic costs may be substantially higher during the mating season than in winter, as suggested by more pronounced body mass loss and increased activity during reproductive periods [68].

The mating season was also characterized by changes in the monkeys’ activity allocations. In the mating season, both sexes significantly increased their time investments in social activities (which encompass mating, aggression and grooming) and moving (Figure 5). The higher percentage of social activities in the mating season at least partly reflects an increase in competitive behaviors. The temporal correlation between mating activity and increased aggression may be driven by selective pressures for both repelling competitors and ensuring fertilization success. While black-and-white snub-nosed monkey breeding units are single-male, multi-female, the proximity to other breeding units offers numerous opportunities for extra-unit mating [40,69,70], thus essentially creating a polygynandrous mating system [71]. The more competitive situation experienced by males in the mating season may contribute to higher energy expenditure, which is associated with substantial body mass loss, as seen in other mammals as well [18,19,21,24]. Furthermore, the degree of body mass loss experienced by males was a direct function of the number of females in their breeding units (Figure 4). Larger ‘harems’ require greater investments in costly mating activities and mate defense, with resulting elevated energy and body mass loss [72,73].

In the mating season, females also significantly allocated more time to social activities and moving, and they reduced their time spent resting (Figure 5). This activity profile may reflect higher energy expenditure and is associated with the observed body mass loss [24,74]. The demands associated with reproductive effort and mating competition may have kept the females ‘on the edge’ and resulted in lost resting opportunities. Competition over mating opportunities increases in social systems where adult sex ratios are strongly biased towards females [75] such as in black-and-white snub-nosed monkey breeding units with a mean male/female ratio of 1:3 (and up to 1:5; Table 1). A behavioral indicator of elevated female–female mating competition is frequent sexual solicitation toward males. In fact, the majority of copulations are initiated by females in this species [42,44] and its congener *R. roxellana* [69]. There is also evidence that in OMUs with a larger number of females solicitation frequency is higher and individual solicitation success is lower [76]. Females are also subjected to sexual interference during mating from co-residing females [63,76,77,78], and such harassment can undermine reproductive outcomes for the victims [76,78]. During the mating season, females actively engage in grooming interactions with males to increase their chances of successful mating [79]. This higher frequency of solicitation can alter the overall activity budget [80] and contribute to the increased moving time and reduced resting time observed during the mating season (Figure 6), leading to higher energy expenditure.

We acknowledge a limitation that the formal analyses are based on a single annual cycle of data. Although our long-term monitoring (2010–2014) and preliminary multi-year analyses suggest consistent seasonal patterns, the single-year dataset may restrict the broader generalizability of our results.

## 5. Conclusions

In this study, we examined seasonal fluctuations in body mass in black-and-white snub-nosed monkeys (*R. bieti*), a high-altitude primate facing dual seasonal challenges: environmental stress in winter and reproductive stress in summer. Body mass peaked in early summer, followed by pronounced loss during the mating season and a less marked decline in winter. While behavioral adjustments helped buffer winter nutritional and thermal constraints, reproductive demands required elevated activity and restricted opportunities for rest and energy intake, resulting in greater body mass loss. Our findings highlight that body mass loss was more pronounced during the mating season than in winter, suggesting that reproductive activity may be associated with relatively higher energetic costs than winter environmental constraints.

## Figures and Tables

**Table 1:** Demographic structure of the study group of black-and-white snub-nosed monkeys in Shangri-la Yunnan Golden Monkey National Park for the period 2010–2013.

Date	Unit	Number				
		Infants	Juveniles	Adult Females	Adult Males	Total
15 March 2013	DGZ OMU	2	3	4	1	10
	YDH OMU	1	4	4	1	10
	HC OMU	1	4	2	1	8
	PG OMU	1	1	1	1	4
	DB OMU	1	3	2	1	7
	BL OMU	0	2	2	1	5
	LHG OMU	0	4	2	1	7
	AMU	-	7	-	5	12
	Total	6	28	17	12	63
15 April 2012	DGZ OMU	3	5	4	1	13
	YDH OMU	1	3	5	1	10
	HC OMU	0	7	4	1	12
	BL OMU	1	4	3	1	9
	XW OMU	1	4	2	1	8
	DHZ OMU	2	5	4	1	12
	SHB OMU	1	0	1	1	3
	LHG OMU	1	4	5	1	11
	AMU	-	6	-	3	9
	Total	10	38	28	11	87

*Note*: Infants are individuals less than one year old; juveniles are individuals older than one year who have not yet reached sexual maturity; adult females are sexually mature (≥six years old); adult males are sexually mature (≥eight years old). OMU = one-male, multi-female unit; AMU = all-male unit.

**Table 2:** Estimates for the fixed effects of the GLMM examining the impact of season on body mass variation in males and females.

Gender	Season	Estimate	Std. Error	t	*p*	CI_2.5%_	CI_97.5%_
Male	(Intercept)	21.12	0.42	50.0	<0.001	20.28	21.94
	Season_Spring_	1.36	0.29	4.68	<0.001	0.79	1.93
	Season_Summer_	1.78	0.27	6.47	<0.001	1.24	2.31
	Season_Winter_	0.01	0.24	0.04	0.97	−0.48	0.49
Female	(Intercept)	10.80	0.25	42.86	<0.001	10.30	11.29
	Season_Spring_	0.97	0.19	5.17	<0.001	0.60	1.34
	Season_Summer_	1.10	0.17	6.38	<0.001	0.76	1.44
	Season_Winter_	0.32	0.17	1.91	0.06	−0.01	0.64

## Data Availability

The original contributions presented in this study are included in the article. Further inquiries can be directed to the corresponding authors.
