# Correction: Li et al. Balancing Reproduction and Survival: Seasonal Body Mass Dynamics in a High-Altitude Primate (*Rhinopithecus bieti*). *Animals* 2026, *16*, 1603

**DOI:** 10.3390/ani16172696

**Published:** 2026-08-31

**Authors:** Yan-Peng Li, Zhi-Pang Huang, Cyril C. Grueter, Xiao-Bin He, Ru-Liang Pan, Xin-Ming He, Gui-Wei Yang, Hua Wu, Liang-Wei Cui, Wen Xiao

**Affiliations:** 1 Institute of Eastern-Himalaya Biodiversity Research, Dali University, Dali 671003, China; liyp@eastern-himalaya.cn (Y.-P.L.); huangzp@eastern-himalaya.cn (Z.-P.H.); 2 School of Life Sciences, Central China Normal University, Wuhan 430079, China; 3 International Center of Biodiversity and Primate Conservation, Dali University, Dali 671003, China; cyril.grueter@anthro.ox.ac.uk (C.C.G.); ruliang.pan@uwa.edu.au (R.-L.P.); 4 Department of Anatomy, Physiology and Human Biology, School of Human Sciences, The University of Western Australia, Perth, WA 6009, Australia; 5 Institute of Human Sciences, School of Anthropology and Museum Ethnography, University of Oxford, Oxford OX1 2JD, UK; 6 Centre of Excellence in Biodiversity and Natural Resource Management, University of Rwanda, Kigali P.O. Box 4285, Rwanda; 7 Centre for Evolutionary Biology, School of Biological Sciences, The University of Western Australia, Perth, WA 6009, Australia; 8 Administration of Baimaxueshan National Nature Reserve, Diqing 674500, China; 795310@163.com (X.-B.H.); hexinming0616@126.com (X.-M.H.); 9 Administration of Gaoligongshan National Nature Reserve in Nujiang, Nujiang 673200, China; ygw918@foxmail.com; 10 Key Laboratory of Minimal Population Wildlife Conservation in Colleges and Universities of Yunnan Province, Southwest Forestry University, Kunming 650224, China; cuilw@eastern-himalaya.cn

There was an error in the original publication [1]. The text and associated reference incorrectly referred to male mountain gorillas and cited Wright et al. (2015), whereas the intended example concerned female Hanuman langurs and should have cited Koenig et al. (1997). The following correction has been made to the second paragraph of the Introduction:

“In Hanuman langurs (*Semnopithecus entellus*), conceptions were concentrated during periods of best female physical condition, suggesting that nutrition influences reproductive timing and conception probability [29].”

Reference [29] has been corrected to the following:

Koenig, A.; Borries, C.; Chalise, M.K.; Winkler, P. Ecology, nutrition, and timing of reproductive events in an Asian primate, the Hanuman langur (*Presbytis entellus*). *J. Zool.* **1997**, *243*, 215–235.

The authors state that the scientific conclusions are unaffected. This correction was approved by the Academic Editor. The original publication has also been updated.
